# PathoFusion: An Open-Source AI Framework for Recognition of Pathomorphological Features and Mapping of Immunohistochemical Data

**DOI:** 10.3390/cancers13040617

**Published:** 2021-02-04

**Authors:** Guoqing Bao, Xiuying Wang, Ran Xu, Christina Loh, Oreoluwa Daniel Adeyinka, Dula Asheka Pieris, Svetlana Cherepanoff, Gary Gracie, Maggie Lee, Kerrie L. McDonald, Anna K. Nowak, Richard Banati, Michael E. Buckland, Manuel B. Graeber

**Affiliations:** 1 School of Computer Science, The University of Sydney, J12/1 Cleveland St, Darlington, Sydney, NSW 2008, Australia; guoqing.bao@sydney.edu.au; 2 Ken Parker Brain Tumour Research Laboratories, Brain and Mind Centre, Faculty of Medicine and Health, The University of Sydney, Sydney, NSW 2006, Australia; raxu4545@uni.sydney.edu.au (R.X.); cloh4889@uni.sydney.edu.au (C.L.); oade2535@uni.sydney.edu.au (O.D.A.); dpie0799@uni.sydney.edu.au (D.A.P.); 3 Department of Neurosurgery, Xuanwu Hospital, Capital Medical University, No. 45 Changchun Street, Beijing 100053, China; 4 St Vincent’s Hospital, Victoria Street, Darlinghurst, NSW 2010, Australia; svetlana.cherepanoff@gmail.com (S.C.); gary.gracie@svha.org.au (G.G.); 5 Department of Neuropathology, RPA Hospital and Brain and Mind Centre, Faculty of Medicine and Health, The University of Sydney, Sydney, NSW 2006, Australia; maggie.lee@sydney.edu.au (M.L.); michael.buckland@sydney.edu.au (M.E.B.); 6 Cooperative Trials Group of Neuro-Oncology (COGNO), Sydney, NSW 1450, Australia; k.mcdonald@braincancerconsultancy.com (K.L.M.); anna.nowak@uwa.edu.au (A.K.N.); rib@ansto.gov.au (R.B.); 7 Brain Cancer Consultancy, Sydney, NSW 2040, Australia; 8 Department of Medical Oncology, University of Western Australia, Perth, WA 6009, Australia; 9 Life Sciences, Australian Nuclear Science and Technology Organisation, Sydney, NSW 2234, Australia; 10 Medical Imaging and Radiation Sciences, Brain and Mind Centre, Faculty of Medicine and Health, The University of Sydney, Sydney, NSW 2006, Australia

**Keywords:** artificial intelligence, bifocal convolutional neural network, CD276, malignant glioma, microvascular proliferation

## Abstract

**Simple Summary:**

We present an open-source AI framework for marking, training, and automated recognition of pathological features in whole-slide scans of diagnostic tissue sections. The integrated system permits high-resolution qualitative as well as quantitative morphological analyses of entire histological slides and harbors significant potential to facilitate the microscopic analysis of complex pathomorphological problems and the simultaneous mapping of immunohistochemical markers in routine slide diagnostics.

**Abstract:**

We have developed a platform, termed PathoFusion, which is an integrated system for marking, training, and recognition of pathological features in whole-slide tissue sections. The platform uses a bifocal convolutional neural network (BCNN) which is designed to simultaneously capture both index and contextual feature information from shorter and longer image tiles, respectively. This is analogous to how a microscopist in pathology works, identifying a cancerous morphological feature in the tissue context using first a narrow and then a wider focus, hence bifocal. Adjacent tissue sections obtained from glioblastoma cases were processed for hematoxylin and eosin (H&E) and immunohistochemical (CD276) staining. Image tiles cropped from the digitized images based on markings made by a consultant neuropathologist were used to train the BCNN. PathoFusion demonstrated its ability to recognize malignant neuropathological features autonomously and map immunohistochemical data simultaneously. Our experiments show that PathoFusion achieved areas under the curve (AUCs) of 0.985 ± 0.011 and 0.988 ± 0.001 in patch-level recognition of six typical pathomorphological features and detection of associated immunoreactivity, respectively. On this basis, the system further correlated CD276 immunoreactivity to abnormal tumor vasculature. Corresponding feature distributions and overlaps were visualized by heatmaps, permitting high-resolution qualitative as well as quantitative morphological analyses for entire histological slides. Recognition of more user-defined pathomorphological features can be added to the system and included in future tissue analyses. Integration of PathoFusion with the day-to-day service workflow of a (neuro)pathology department is a goal. The software code for PathoFusion is made publicly available.

## 1. Introduction

Image analysis is a driver for the development of artificial intelligence (AI) applications and great progress has been made in recent years following the introduction of convolutional neural networks (CNNs) [1,2]. Morphological disciplines such as pathology are likely to benefit from the development of specific AI, and publications on the use of CNNs in pathology as well as neuropathology have begun to appear [3,4]. An AI-supported workflow in pathology can already be envisioned.

Immune checkpoint markers are of special interest in cancer research because they may represent powerful new therapeutic targets, as suggested by the significant progress made, especially in the field of melanoma [5,6,7,8,9]. Microvascular proliferation is one of the essential hallmarks of glioblastoma [10]. It is a more specific and reliable sign of malignancy than necrosis, another key morphological feature that distinguishes glioblastoma from WHO grade III glioma. While it is not only endothelial cells that constitute the cellular substrate of microvascular proliferation [11,12,13], recent reports have shown that CD276 (B7-H3), an immune checkpoint marker of prognostic significance [14,15,16], is strongly expressed by abnormal endothelial cells in various cancers including glioblastoma (Figure S1) [14,17,18,19]. The inclusion of immunohistochemical (IHC) data has become a standard for cancer diagnostics and research. 

Given the complex morphological characteristics of human tissue biopsies, their analysis requires an ever-increasing amount of computing resources and advanced learning algorithms. The arrival of more advanced deep CNNs in recent years has made the analysis of whole-slide tissue sections possible. For example, deep residual networks (ResNet) [20] introduced skip connection to eliminate singularities and alleviate the vanishing gradient problem, which enables training of CNNs that are hundreds and even thousands of layers deep. One of the widely used ResNet models is ResNet-50, which is 50 layers deep and has been used as the reference when comparing network performances. Even more complex architecture such as Xception [21] integrated skip connection with depth-wise separable convolution and achieved superior performance in comparison to its predecessors. 

On the basis of advanced deep learning models, we have developed an integrated system, termed PathoFusion, which is an AI-based platform for marking, training, and recognition of pathological features in whole-slide images (WSIs). We have used tissue sections obtained from glioblastoma cases to evaluate the system. Adjacent tissue sections were processed for hematoxylin and eosin (H&E) staining and IHC (CD276), respectively, and scanned. PathoFusion is designed to meet three goals: (i) efficient training of convolutional neural networks to recognize key pathomorphological features in routine H&E-stained, scanned histological slides; (ii) improved model generation and increased effectiveness of feature recognition, thus requiring fewer physical cases than conventionally needed for neural network training; and (iii) establishing a method that allows the inclusion of immunohistochemical (IHC) data in the automated analysis given the great importance of immunohistochemistry in contemporary slide-based analyses.

## 2. Results

### 2.1. Recognition of Morphological Features and Associated Immunoreactivity

**Figure 1:**
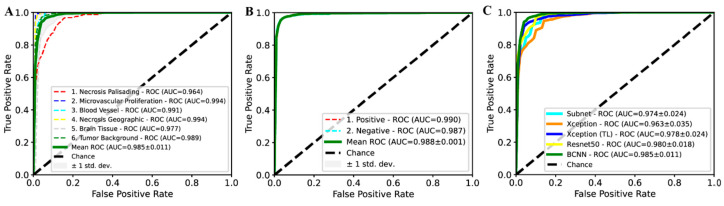
Receiver operating characteristic (ROC) and area under the curve (AUC) performance on test data. ROC/AUC performance of the bifocal convolutional neural network (BCNN) for recognition of hematoxylin and eosin (H&E) (**A**) and immunohistochemical (IHC) (**B**) features. (**C**) Comparison of ROC/AUC performance of BCNN with state-of-the-art deep learning methods.

A bifocal convolutional neural network (BCNN) was designed and integrated into the PathoFusion platform to make use of bifocal image pairs as described in the methods section. The BCNN was trained on full training/cross-validation data and was subsequently evaluated on H&E and IHC test data, respectively. The predicted class of each paired image patch was assessed against expert marking (ground truth). As shown in Figure 1A,B, PathoFusion reliably identified morphological features (area under the curve (AUC) of 0.985 ± 0.011, precision of 94.4%, and sensitivity of 94.7%) and immunoreactivities (AUC of 0.988 ± 0.001, precision of 96.2%, and sensitivity of 96.1%), respectively, enabling correlation of the tissue expression of CD276 with diagnostic morphological features. 

**Figure 2:**
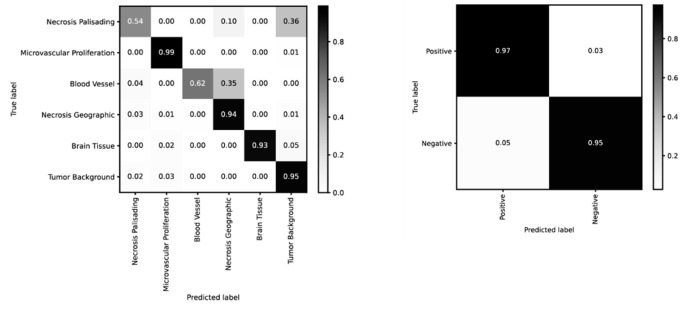
Confusion matrices for recognition of H&E (left) and IHC (right) features (BCNN; normalized).

PathoFusion achieved higher prediction performance for microvascular proliferation (AUC of 0.994) and geographic necrosis (AUC of 0.994) than palisading necrosis (AUC of 0.964); Figure 1A. This result is consistent with a real-world scenario because the latter is also more difficult to identify for human observers. Therefore, the six selected pathomorphological features are currently not recognized at the same level. This is also apparent from the confusion matrix in the left panel of Figure 2. In the much simpler binary case of immunostained sections, PathoFusion achieved the same high performance in recognition of both positive (AUC of 0.990) and negative (AUC of 0.987) tissue areas (Figure 1B and Figure 2 (right panel)) as expected.

As shown in Figure 1C, the BCNN integrated into our framework achieved higher performance than popular state-of-the-art deep learning models, including ResNet-50, Xception and transfer learning (TL)- [22,23,24] based Xception (pretrained on ImageNet [25]). Importantly, however, the BCNN utilizes only half of the number of network parameters compared to ResNet-50 and Xception, suggesting that our BCNN is a more efficient neural network for the analysis of histopathology images. In addition, the BCNN achieved superior performance compared to its subnet implementation (Figure 1C), which demonstrated the effectiveness of the bifocal design. An analysis of the mechanisms underlying such a performance gain is beyond the scope of this work and has been dealt with elsewhere [26].

### 2.2. Analysis of Entire Histological Sections

**Figure 3:**
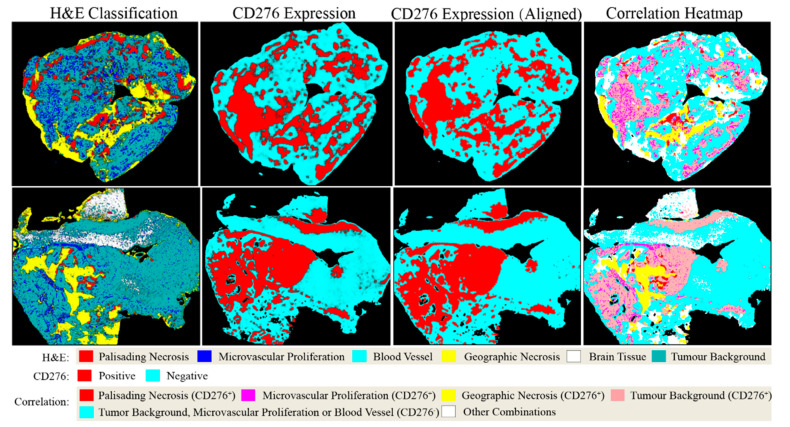
Predicted heatmaps of test cases showing distribution of machine-recognized features. First column: heatmaps showing the distribution of pathomorphological features predicted from scanned H&E whole-slide images; second and third columns: heatmaps of the same cases showing the distribution of CD276 immunoreactivity before and after image registration, respectively; last column: CD276 expression mapped to pathomorphological features following image registration. Note: Probability heatmaps in the second column were generated using prediction probabilities as weighting factors for pseudo-color mapping, which were then converted into classification heatmaps before image registration (third column) and image fusion (fourth column).

**Figure 4:**
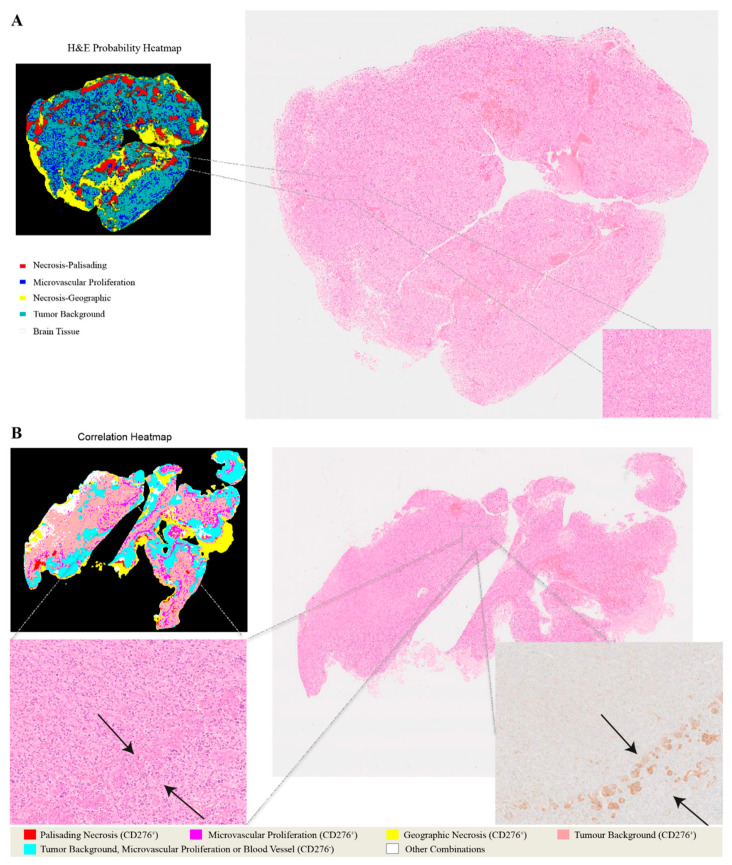
Heatmaps illustrating the relationship between H&E images and CD276 immunohistochemistry. (**A**) Predicted probability heatmap of key morphological features in relation to the corresponding H&E image. (**B**) Correlation heatmap and the spatial overlaps between H&E-stained morphological structures and CD276 immunoreactivity.

**Figure 5:**
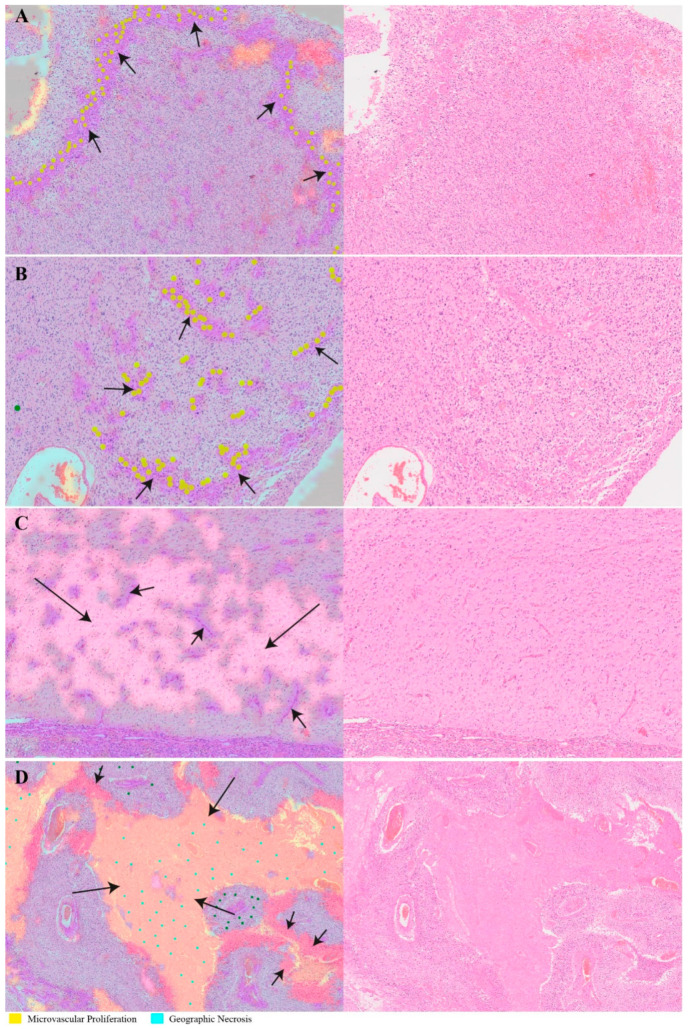
Transparent overlays (left column) of predicted feature distribution heatmaps on original whole-slide test images (right column). Images and crops from the images were randomly selected. (**A**,**B**) Machine-recognized tissue areas (arrows; predicted to contain microvascular proliferation) closely match the expert marking (annotated yellow dots). (**C**) A machine-recognized tumor-infiltrated brain tissue sample (long arrow) also shows incipient microvascular proliferation (short arrows). (**D**) Another illustration of recognized pathomorphological features showing different subcategories of tissue necrosis: geographic necrosis (long arrows) and palisading necrosis (short arrows).

The capability of PathoFusion in tile-level recognition formed the basis for the automated analysis of entire histological sections. As shown in Figure 3, adjacent whole-slide H&E and IHC images were recognized by the PathoFusion framework and converted into heatmaps which visualize the tissue distribution of the six chosen morphological features (first column) as well as corresponding immunoreactivity (subsequent two columns). Sample matching signals of detected microvascular proliferation in H&E whole-slide tissue sections are illustrated in Figure 4A. The predicted heatmaps can be transparently overlaid onto the corresponding H&E-stained tissue sections to allow direct visual inspection and comparative checking (Video S1). The examples shown in Figure 5 demonstrate that the BCNN-recognized morphological features closely match the neuropathological features delineated by expert marking. Certain morphological features of which subcategories exist will benefit from additional refinement. Quantitative analysis of sample cases revealed that microvascular proliferation accounted for a sizeable fraction of the H&E heatmaps in line with microscopic appearance (Table S1).

The literature on CD276 tissue staining is limited. We have checked the antibody labeling using a second automated stainer system employing both tumor and non-tumor tissue for control (we compared Leica vs. Ventana). The staining of abnormal tumor blood vessels was strong in both cases. However, it appears that the Ventana method may be more sensitive and there is some interesting immunolabeling outside blood vessels with the latter. Clearly, the additional result deserves further scrutiny.

### 2.3. Fusion of Bimodal Neuropathological Images

In addition to recognizing key pathomorphological features in glioblastoma, PathoFusion was shown to be capable of simultaneously mapping the expression of immunoreactivity (CD276 in this work) in adjacent tissue sections, facilitating the interpretation of complex immunohistochemical staining results. The correlative overlap between morphological features and immunoreactivity in the bimodal whole-slide images was visualized through image fusion of the corresponding predicted heatmaps. As shown in Figure 3, the H&E-based heatmaps (first column) were fused with the aligned IHC heatmaps (third column), which resulted in correlation heatmaps (last column) representing feature overlap of the two modalities. For example, the color magenta represents matching signals in tissue areas harboring both microvascular proliferation and associated CD276 immunopositivity. The close association between CD276 and microvascular proliferation in (glioblastoma multiforme) GBM biopsies was clearly demonstrated, permitting qualitative as well as quantitative tissue analyses. Matching signals between microvascular proliferation and CD276 immunopositivity are further illustrated in Figure 4B. 

Quantitative and correlation analyses confirmed that microvascular proliferation had a significantly higher (specific) CD276 expression compared to visually negative normal blood vessels (Table S2 and Figure S1). However, a detailed analysis of CD276 expression is beyond the scope of this paper.

## 3. Discussion

Morphological diagnostic work in pathology, and especially in neuropathology—given the great structural complexity of the nervous system—has elements of an art. In fact, “painting with words” is a skill taught for the writing of useful microscopic reports that convey a synthesis of complex visual information, requiring both creativity and imagination for their generation. It may seem counterintuitive, therefore, that an AI should be capable of acquiring the equivalent of typical human skills, but similar developments are occurring in other fields, in- and outside of medicine—in software programming and even music, to name a few.

The PathoFusion framework introduced here provides new tools that may facilitate the transfer of pathomorphological feature detection knowledge to machines. The framework is suited for the analysis of pathomorphological hallmarks in whole histological slides, as also demonstrated in Video S1, allowing the development of a specific (neuro)pathological AI. Both marking and training times can be reduced when using the integrated system, accommodating the time constraints relevant to busy clinical consultants. Expert input is the key limiting factor, since the marking of pathological features (“ground truth”) cannot be delegated to less well-trained observers. On the other hand, a comparatively small cohort of human biopsies was required to carry out the necessary training, which resulted in high testing performance. Being able to train neural networks effectively on the basis of a relatively small number of cases should be useful in many scenarios. 

The histological slides used in pathology and neuropathology can be scanned and converted into digital images. Those images contain an exceptionally rich variety of structural detail and staining patterns. The file size of these images is extremely large (gigabytes), which makes them very challenging to process using conventional methods. However, in all other respects, the images used here are comparable to the digital photographs that are widely employed and have become a predominant focus of AI research. CNNs have been found to be particularly suited for the analysis of images.

A number of earlier studies have demonstrated the utility of repurposing CNN models pretrained on natural image collections such as ImageNet for medical image analysis [27,28]. This approach is referred to as transfer learning and has worked well in some cases where a close relationship exists between the source and target domains, but failed in other instances where knowledge learned from the source domain was less general across sub-domains [29,30,31]. However, our study obviates the need for transfer learning through the use of a dual-path CNN model utilizing bifocal image tiles as input. Our model achieved better recognition performance than popular deep learning models, including ResNet-50 and Xception, pretrained on ImageNet, thus eliminating the need for resource-intensive pretraining.

The prognostic marker CD276 was chosen as a proof-of-concept example, allowing our AI system to validate its recognition ability autonomously by producing a fusion heatmap that demonstrates the overlap between a morphological feature, microvascular proliferation, and CD276 immunoreactivity. The specific task of mapping CD276 to a subset of blood vessels, and endothelial cells of abnormal tumor blood vessels in particular, can also be performed by a human observer, which is why this marker was chosen for machine recognition because a widely accepted feature is more convincing. A human observer may even be able to perform systematic recognition of a morphological feature in an entire histological section, but it would be a very tedious and time-consuming exercise. Interestingly, CD276 labeling in the present study was strong in tumor vasculature, as expected, but not limited to it when using the Ventana automated tissue staining system. We believe that the CD276 labeling noted when using this staining system deserves further analysis, which is beyond the scope of this manuscript. Extravascular CD276 labeling, which appeared to be partly cellular, is very interesting with regard to the function of CD276 as an immune checkpoint molecule.

Following training, our BCNNs reliably identified the key morphological features that they had been trained to recognize with AUC performances of 0.985 and 0.988 on H&E and immunohistochemical images, respectively. This formed the basis for the ability to correlate the occurrence of individual pathomorphological features with the tissue expression of CD276. Notably, the close association between CD276 and microvascular proliferation in GBM was faithfully reproduced and visualized by the heatmaps, permitting high-resolution (40× primary magnification) qualitative as well as quantitative morphological analyses of complete histological sections. The method presented here allows quantification of the occurrence of key morphological features and simultaneous matching of immunoreactivities (or other molecular histological data such as in situ hybridization results) to those features. This has not been possible before and may harbor significant potential for brain mapping projects (e.g., Allen Atlas). Another important quality of the PathoFusion framework consists of the independence of its prediction and fusion processes, which do not require human intervention once the BCNN model has been properly trained. The framework is expected to provide a comparable performance on other tumor types and also on non-neoplastic pathological lesions, provided that a qualified observer (experienced consultant) performs the marking, thus establishing the relevant ground truth which is necessary for CNN training. 

There are several methods available to facilitate slide diagnostics—for example, Digital Slide Archive is a web-based interactive system for annotating whole-slide histopathology images which shows a similarity in function to the labeling module of PathoFusion; Cytomine is a machine learning-based open-source platform for collaborative analysis of multi-gigapixel images, but it requires users to provide customized scripts for image analysis. Distinct from Digital Slide Archive and Cytomine, Digipath is the product of a company that provides hardware (e.g., a scanner) as well as online training courses. Compared to all of these, PathoFusion is a light-weight automated framework for the analysis of multi-modal histopathology images, which provides functions ranging from annotation, training and whole-slide image detection to cross-modality quantitative analysis. PathoFusion is also flexible and can be integrated in existing annotation systems, such as Digital Slide Archive, or enhance existing hardware solutions, e.g., a scanner that is capable of detecting tissue structures as well as producing heatmaps for abnormalities.

The present study has the following limitations. Firstly, we acknowledge that training for the recognition of some pathomorphological features could be improved further by creating more subcategories as well as carefully defining any similar-appearing morphologies (mimics that can cause diagnostic pitfalls), e.g., geographic necrosis with and without bleeding, and palisading necrosis vs. tumor cell palisades without necrosis. Secondly, many additional pathomorphological features could be added to the training in order to be fully prepared for possible confounding pathological signs of unrelated but co-occurring diseases. Thirdly, the quantifications performed in this study were carried out for formal reasons and are not necessarily biologically understood. For instance, some of the novel CD276 immunohistochemical results such as lower-level CD276 expression outside of the tumor vasculature when using the Ventana system deserve additional study.

## 4. Materials and Methods 

### 4.1. Clinical Cases

Paraffin sections of 34 WHO grade IV glioma samples, provided by the Australian Genomics and Clinical Outcomes of Glioma (AGOG) tissue bank, were used for this study (University of Sydney Human Ethics Committee Project number 2016/027). Paraffin sections were stained with H&E and scanned at 40X magnification using an Olympus VS-120 scanner. Adjacent sections were processed for CD276 immunohistochemistry at St. Vincent’s Hospital, Sydney, and at the Department of Neuropathology of Royal Prince Alfred Hospital, respectively.

### 4.2. PathoFusion Framework

#### 4.2.1. Expert Marking and Datasets

An in-house labeling website was developed to facilitate marking of morphological features by a neuropathology consultant (Figure S2). The website forms part of the PathoFusion framework, which, in addition to conventional web server hard- and software, makes use of a laptop computer for manually marking relevant structures. For the initial training and subsequent testing of the BCNN, six typical morphological features were chosen and marked by a consultant neuropathologist (M.B.G.) following WHO criteria [10] where appropriate: palisading necrosis (I), microvascular proliferation (II), histologically normal-appearing blood vessels (III), geographic necrosis (IV), brain tissue (V), and tumor background (extensive diffuse infiltration of brain tissue by glioma cells, (VI). We used approximately 850 marking dots on average in each whole-slide image to cover six typical pathomorphological features. Each marking dot used for labeling a relevant feature provided the tissue section co-ordinates for a whole or part of a specific morphological feature of interest.

**Figure 6:**
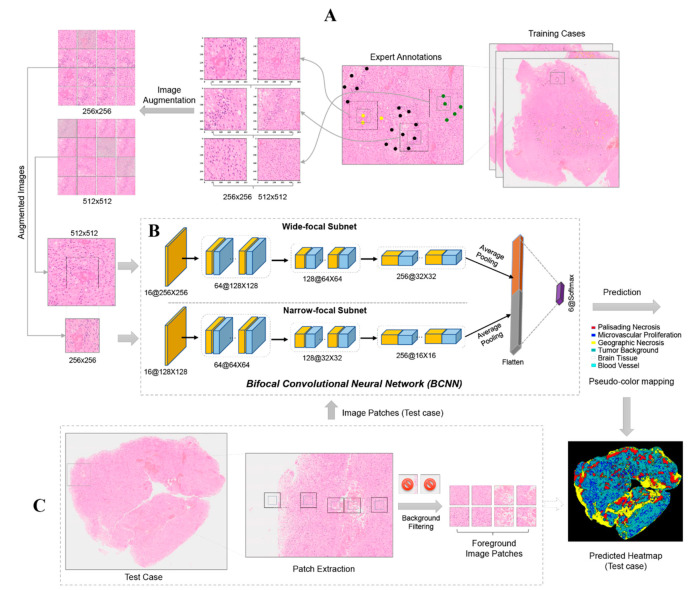
Overview of the PathoFusion framework for recognition of pathological features. Upper panel (**A**): Typical pathomorphological features are manually marked on whole-slide scans with the help of a labeling website (Figure S2). Three examples are shown: palisading necrosis (black dots), microvascular proliferation (yellow dots), and tumor background (defined as extensive diffuse infiltration of brain tissue by glioma cells, green dots). Based on the marking coordinates, 256 × 256- and 512 × 512-pixel paired image tiles are extracted (**A**, center panel), followed by image augmentation to expand the training dataset. In the next step (**B**), paired augmented image tiles are fed into the BCNN for supervised learning. (**C**) Test cases are used to evaluate recognition performance. The distribution of key morphological features is obtained for entire scanned slides as illustrated by the prediction (probability) heatmap. Note: the pseudo-colors of the heatmap are different from the colors used for marking.

In brief, a total of 58,526 paired image tiles (sizes of 512 × 512 and 256 × 256 pixels, respectively) were extracted from the 34 H&E whole-slide scans. Every paired image tile was based on one of 29,106 individually marked coordinates (epicenters for extraction) for the recognition of the six selected morphological features. In contrast, the marking of IHC images included only two criteria, i.e., the presence or absence of brown diaminobenzidine-peroxidase reaction product indicating antibody binding, which was based on pixel intensity. A total of 20,644 paired image tiles were extracted from CD276 slide scans. Furthermore, 6648 paired image tiles from 10% of the cases were employed for testing and 51,878 paired image tiles from the remaining scans were used for training and cross-validation. Training and testing data were extracted from different cases and there was no overlap between them. Details of the marking and extraction procedure are illustrated in Figure 6A.

#### 4.2.2. Bifocal Convolutional Neural Network (BCNN)

The BCNN employed in the PathoFusion framework possesses two input paths that accept one narrow-focus image tile and one paired wide-focus image tile simultaneously. This network is designed to capture both index and contextual feature information from shorter and longer image tiles, respectively. This is analogous to how a human observer works, identifying a feature in the tissue context using first a narrow and then a wider focus (hence, bifocal). Thus, the BCNN consists of two convolutional subnets, one module for feature concatenation, and a layer for classification (Figure 6B). Each subnet has a structure similar to ResNet-26 [20] but without skip connections [20]. Detailed information and mathematical formulas characterizing the network are beyond the scope of this paper and have been described elsewhere [26]; however, core information has been added as supplementary material.

#### 4.2.3. Recognition of Morphological Features and Associated Immunoreactivity

At the training stage, image augmentation including rotation, contrast, and sharpness adjustments was randomly applied to bifocal image tiles before they were fed into the BCNN model (Figure 6B). The actual training input was therefore diversified and expanded greatly (*n*^2^ times) compared to conventional methods (*n* times) (left panel, Figure 6A), where *n* is the number of augmentation types. Following training of the model, paired image tiles were extracted from the upper L to bottom R of the whole-slide test images with a stride of 50 pixels (middle panel, Figure 6C). Each foreground image patch pair was classified by the model and assigned to one of the expert-defined morphological feature or immunoreactivity categories—for example, microvascular proliferation and immunopositive expression. The prediction results were converted into feature heatmaps using pseudo-colors (bottom R, Figure 6). It is worth noting that no augmentation was involved at the recognition stage.

#### 4.2.4. Method for Fusing Bimodal Histological Images

**Figure 7:**
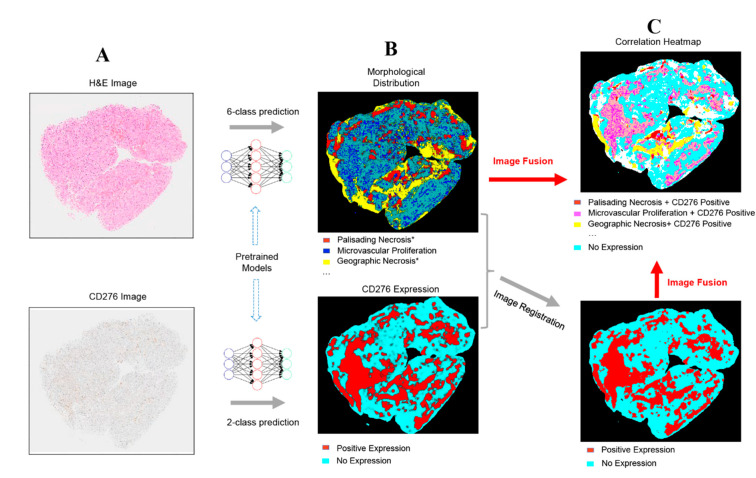
Overview of the PathoFusion framework for mapping of immunohistochemical data to morphological features. Whole-slide histopathology images of different modalities (**A**) are processed by the BCNN and used to generate heatmaps, showing the predicted distribution of morphological features and CD276 expression patterns, respectively (**B**). The immunohistochemical heatmap is then aligned with the H&E heatmap using image registration (**C**, lower panel). The correlation between the two modalities can be visualized through fusion of the corresponding heatmaps. Each color in the correlation heatmap (**C**, upper panel) indicates an overlap between the two modalities. The asterisks indicate that further marking and training are required to differentiate subcategories of palisading and geographic necrosis, respectively (see text for further explanation). The overlap between microvascular proliferation and CD276 immunopositivity is of special interest (magenta color in the upper panel of **C**).

An image fusion method was developed to visualize the correlation (spatial overlap) between recognized morphological features and immunohistochemical staining results (both were BCNN predictions) using bimodal images. In order to achieve this, H&E and IHC whole-slide images were first processed by the pretrained BCNN model (Figure 7A) and then converted into heatmaps (Figure 7B). Since the corresponding H&E and IHC images were obtained from adjacent tissue sections of the same biopsy, an image registration algorithm [32], making use of feature point correspondences to establish matching signals, was used to align (pair) the IHC heatmap with the corresponding H&E feature heatmap to allow subsequent combined analysis (Figure 7C). The aligned heatmaps were then merged to create the fusion heatmap, and each color in the final fused correlation heatmap represents a distinct spatial association between one of the morphological features and immunoreactivity.

#### 4.2.5. Quantitative Analysis

The area percentage for each of the six morphological features within H&E whole-slide images was first determined based on classification heatmaps denoted as $P_{he(i)}$. Accordingly, $P_{ihc(i)}$ represents the percentage of immunohistochemically positive and negative areas in relation to whole-slide IHC image scans. Next, the percentage of CD276 positivity for each of the diagnostic morphological features was calculated based on the correlation heatmaps as $P_{i}=I_{i}/F_{he(i)}$,where $I_{i}$ represents the intersection between a morphological feature i and an immunopositive area, while $F_{he(i)}$ denotes the area taken by a morphological feature i.

#### 4.2.6. Data Availability and Experimental Reproducibility

The dataset and the source code used in this study have been released to the public at https://github.com/guoqingbao/Pathofusion. The source code written for our pathology image marking website will be provided for non-commercial and research purposes upon request.

## 5. Conclusions

Taken together, our results demonstrate that routine histopathological sections from a comparatively small number of cases can be used to train a BCNN effectively. Applied to entire histological sections, PathoFusion has the potential to facilitate the microscopic analysis of complex immunohistochemical markers and H&E-stained tissues in real-world scenarios. In addition, color normalization of histopathology images [33] may make our method more widely applicable, i.e., when dealing with stained images from different laboratories where variations in color and intensity of the images exist. We are working on improving the capabilities of PathoFusion further by adding more feature definitions and refining the categorization of complex pathomorphological entities such as necrosis subtypes. Integration of the system with the workflows of a neuropathology or pathology department is a goal. In the future, a fully developed expert AI system may especially benefit patients in geographic areas that do not have local access to specialist pathological services such as neuropathology.

## Data Availability

The datasets for image patches presented in this study are openly available in https://github.com/guoqingbao/Pathofusion/tree/master/data; Data of the tissue sections are available from the authors.

## Supplementary Materials

The following are available online at https://www.mdpi.com/2072-6694/13/4/617/s1, Figure S1: CD276 immunopositivity of glioblastoma vasculature., Figure S2: Pathology image labeling module., Table S1: Percentage of each diagnostic feature within whole-slide H&E and IHC images (sample)., Table S2: Percentage of CD276 positivity for each diagnostic morphological feature (sample)., Video S1: Video demonstration of the AI framework for detection of cancerous features in whole-slide tissue sections.
