# Supplementary material for: PathoFusion: An Open-Source AI Framework for Recognition of Pathomorphological Features and Mapping of Immunohistochemical Data
Source: Cancers (Basel). 2021 Feb 4;13(4):617. doi: 10.3390/cancers13040617 (PMC7913958; doi:10.3390/cancers13040617)
Supplement: Supplementary file 1 [file cancers-13-00617-s001.pdf]

cancers

MDPI

Article

Supplementary Material: PathoFusion: An Open-Source AI Framework for Recognition of Pathomorphological Features and Mapping of Immunohistochemical Data

Guoqing Bao [1], Xiuying Wang [1,*], Ran Xu [2,3], Christina Loh [2], Oreoluwa Daniel Adeyinka [2], Dula Asheka Pieris [2], Svetlana Cherepanoff [4,5], Gary Gracie [4], Maggie Lee [5], Kerrie L. McDonald [6,7], Anna K. Nowak [6,8], Richard Banati [6,9,10], Michael E. Buckland [5,6] and Manuel B. Graeber [2,*]

Neural network design. The bifocal convolutional neural network (BCNN) introduced here has two input paths that accept one narrow-focus image tile (X, $X \in \mathbb{R}^{C\times H\times W\times N}$) and another paired wide-focus image tile (X') simultaneously. The narrow-focus patch contains a view limited to the actual structure of interest, whereas the wide-focus patch not only contains the relevant structure of interest as image content, but additional information on the tissue context of the structure of interest is included as well.

For a given narrow-focus input X, where H and W are spatial dimensions, C and N are the input and output channel dimensions. The paired wide-focus input X' has the same centroid as X, and with a spatial dimension of and $W * \alpha$ and $H * \alpha$, where α is the integer factor to explore. The output of the network is defined as the following:

$$\bar{y} = softmax(concat(G(y), G(y'))) \quad (1)$$

where, G is the Global Average Pooling layer; y and y' are the feature maps that simultaneously generated by the two convolutional branches and defined as:

$$y = relu(x^r) = relu(\phi(\sum_{i=1}^{k^2} w_{ij}^r \cdot x_i^{r-1} + b_j^r)) \quad (2)$$

In other words, y is a product after applying ReLU activation function on x^r, which is the final convolutional output of X and X' in each network branch (output of last convolutional layer r); x^r (also denoted as ϕ) is a stack of convolutional output for each kernel j ($j <$ N); w represents the kernel weights, k is the kernel size, and b denotes biases.

Specifically, the neural network was implemented in the following way: The BCNN consists of two convolutional subnets, one feature concatenation module, and a classification layer. Each subnet is 26-layers deep, has a structure similar to ResNet-26, which consists of a convolutional layer with an output feature map size of 16, three convolutional blocks with the output feature map size ranging from 64 to 256, and one average pooling layer, but without skip connections. Each convolutional block has 8 consecutive convolution/batch normalization pairs. The first convolutional layer has a stride of 2, which downsamples the input image and greatly reduces the amount of computation for the subsequent layers. To make the network more computationally efficient, each convolutional block also introduced a stride of 2 in its first layer, which further reduces the image size from one block to another. The ReLU activation function is applied to each convolutional output for introducing non-linearities to the network. The Softmax function was not explicitly used in the last layer of the network since it is already incorporated in the PyTorch cross-entropy loss function which we adopted for this study. The network was trained to read the paired bifocal image tiles simultaneously.

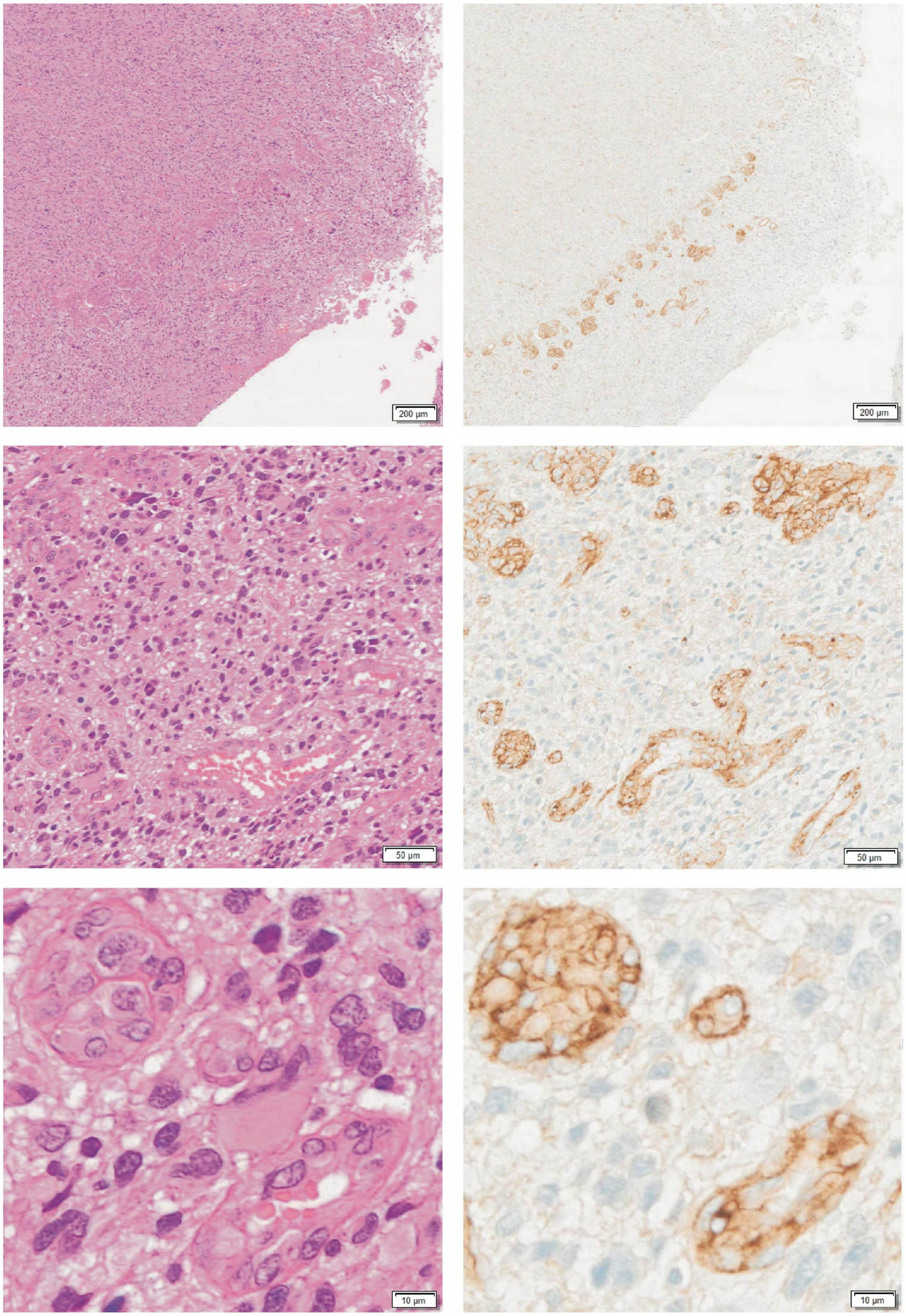

Figure S1. CD276 immunopositivity of glioblastoma vasculature. Adjacent paraffin sections stained for hematoxylin and eosin (left column) and immunohistochemically labeled for CD276 (right column), respectively. Corresponding tissue areas illustrating CD276 immunoreactivity of microvascular proliferation and specifically of abnormal endothelial cells are shown at increasing magnification.

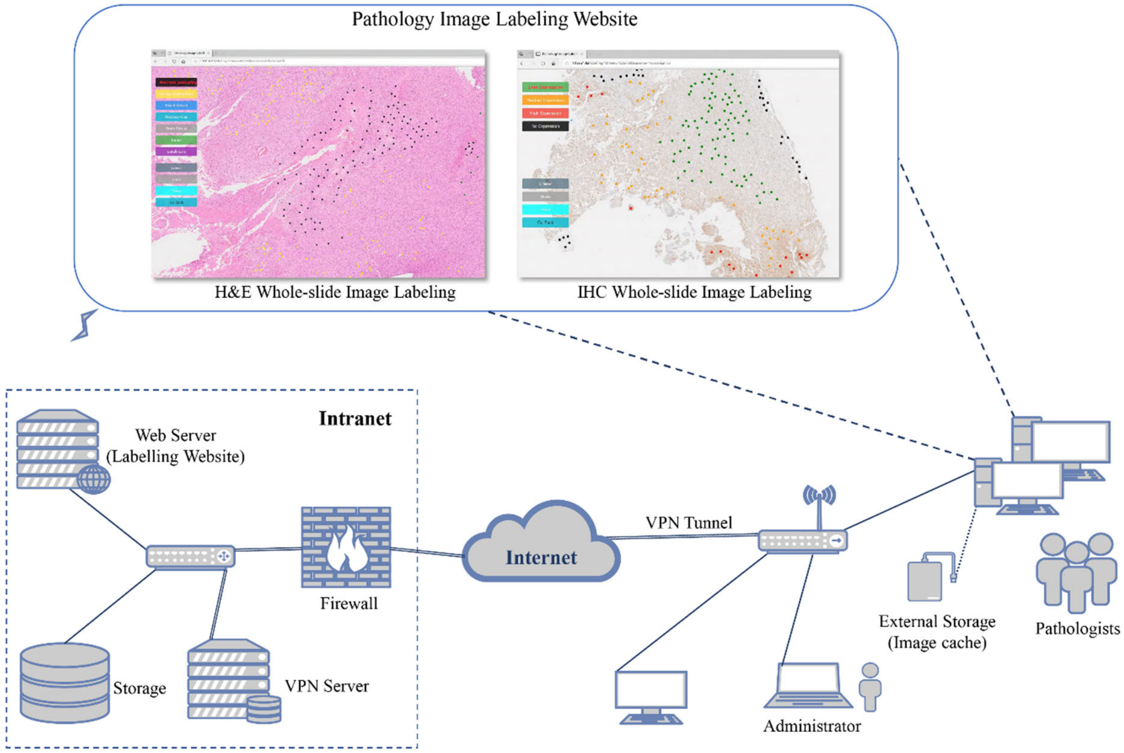

Figure S2. Pathology image labeling module. A website was designed and set up on the institutional intranet for marking pathomorphological features in scanned histopathological slides. The marking was performed by a consultant neuropathologist. A neuropathologist first defines the relevant feature categories for the regions of interest (ROIs) in the backend system, e.g., six types of H&E morphological features in this piece of work. Via the website, the consultant is able to zoom in on the whole-slide H&E and IHC images and mark the different ROIs with color dots. Different colored dots represent the categories that have been predefined in the backend system. The gigabyte-sized image scans are precached on a local computer and the website redirects its image content to the local cache in order to speed up image loading. Therefore, only labeling coordinates need to be uploaded.

Table S1. Percentage of each diagnostic feature within whole-slide H&E and IHC images (sample).

NO.	$P_{he(1)}$	$P_{he(2)}$	$P_{he(3)}$	$P_{he(4)}$	$P_{he(5)}$	$P_{he(6)}$	$P_{ihc(1)}$	$P_{ihc(2)}$
I	12.3	13.4	2.6	18.9	0.2	52.7	37.2	62.8
II	2.0	7.1	1.7	15.8	11.2	62.1	34.6	65.4
III	2.4	13.4	2.8	14.1	4.6	62.8	73.5	26.5
IV	8.8	7.3	2.3	20.9	17.0	43.6	60.5	39.5
V	3.3	5.4	3.1	45.1	0.3	42.7	60.3	39.7

$P_{he(1\text{-}6)}$: Area percentage of detected palisading necrosis (1), microvascular proliferation (2), histologically normal-appearing blood vessels (3), geographic necrosis (4), brain tissue (5), and tumor background (extensive diffuse infiltration of brain tissue by glioma cells, 6), in the respective histological sections. $P_{ihc(1\text{-}6)}$: percentage of detected CD276-positive (1) and -negative (2) expression in the entire histological section. Microvascular proliferation ($P_{he(2)}$) and geographic necrosis ($P_{he(4)}$) accounted for a large fraction of the H&E heatmaps when not considering tumor background ($P_{he(6)}$).

Table S2. Percentage of CD276 positivity for each diagnostic morphological feature (sample).

NO.	P_1	P_2	P_3	P_4	P_5	P_6
I	22.5	59.8	25.4	34.0	36.2	36.8
II	47.0	48.5	23.5	36.2	10.3	32.2
III	62.5	77.4	59.8	77.0	93.8	70.7
IV	53.1	45.9	51.8	54.9	70.4	62.9
V	77.9	91.9	69.7	29.5	37.1	88.5
Avg.	52.6	64.7	46.0	46.3	49.6	58.2

$P_{(1\text{-}6)}$: percentage of detected CD276 positivity in palisading necrosis (1), microvascular proliferation (2), histologically normal-appearing blood vessels (3), geographic necrosis (4), brain tissue (5), and tumor background (extensive diffuse infiltration of brain tissue by glioma cells, 6).

Video S1. Video demonstration of the AI framework for detection of cancerous features in while-slide tissue sections.

Please refer https://github.com/guoqingbao/Pathofusion/tree/master/demo
